# Role of life events in the presence of colon polyps among African Americans

**DOI:** 10.1186/1471-230X-13-101

**Published:** 2013-06-12

**Authors:** Hassan Ashktorab, Hassan Hassanzadeh Namin, Teletia Taylor, Carla Williams, Hassan Brim, Thomas Mellman, Babak Shokrani, Cheryl L Holt, Adeyinka O Laiyemo, Mehdi Nouraie

**Affiliations:** 1 Cancer Center and Department of Medicine, Howard University College of Medicine, 2041 Georgia Avenue, N.W. Washington, D.C. 20060, USA; 2 Department of Pathology, Howard University, college of Medicine, Washington, D.C., USA; 3 Department of Behavioral and, Community Health, Center for Health Behavior Research, School of Public Health, University of Maryland, College Park, Maryland, USA

**Keywords:** Stress, Life events, Colon adenoma, African Americans

## Abstract

**Background:**

African Americans have disproportionately higher incidence and death rates of colorectal cancer among all ethnic groups in the United States. Several lifestyle factors (e.g. diet, physical activity and alcohol intake) have been suggested as risk factors for colorectal cancer. Stressful life events have also been identified as risk factors for colorectal cancer. The association between stressful life events and colon polyps, which are precursors of colorectal cancer, has yet to be determined.

We aimed to evaluate the relationship between stressful life events and the presence of colon polyps and adenomas in African American men and women.

**Methods:**

In this cross-sectional study, 110 participants were recruited from a colon cancer screening program at Howard University Hospital. Participants completed an 82-item Life Events Questionnaire (Norbeck 1984), assessing major events that have occurred in the participants’ life within the past 12 months. Participants also reported whether the event had a positive or negative impact. Three scores were derived (total, positive, and negative).

**Results:**

Total life events scores were higher (Median [M] = 29 and Interquartile range [IQR] = 18-43) in patients with one or more polyps compared to patients without polyps (M, IQR = 21,13-38; P = 0.029). Total, positive or negative Life Events scores did not differ significantly between normal and adenoma patients. Total, negative and positive Life Events scores did not differ between patients who underwent diagnostic colonoscopy (symptomatic) and patients who underwent colonoscopy for colon cancer screening (asymptomatic) and patients for surveillance colonoscopies due to a personal history of colon polyps. Linear regression analysis indicated that male gender is associated with 9.0 unit lower total Life Events score (P = 0.025).

**Conclusion:**

This study suggests that patients who experienced total life events may be at higher risk of having colon polyps and adenomas which indicates an association between stress and the development of colorectal polyps.

## Background

Colorectal cancer (CRC) is the the second leading cause of cancer related deaths in the United States [1]. Prevalence of CRC increases with age [2,3] and is highest during the sixth decade of life. Males have higher burden of disease compared to females [3]. African Americans also have a high incidence of CRC. Apart from age, sex, and ethnicity; lifestyle factors such as obesity, smoking, high fat diet, and physical inactivity increase the risk of CRC [4].

Life events and accompanying psychological and behavioral reactions frequently have an impact on people’s daily lives and are believed to predispose them to diseases [5,6]. Observational studies have established that stressful life events, often defined as an accumulation of ordinary life events or major events such as bereavement, increase the risk of mental disorders [7,8], acute infections such as the common cold [9], and mortality [10]. Life events have also been suggested to contribute to other diseases, including cardiovascular diseases [11,12], asthma[13], and rheumatoid arthritis [14]. Psychosocial stress, through its potential influence on biological processes, has also been associated with the onset and progression of certain medical conditions, including cancer [12,15-18]. Little is known, however, about potential associations between psychosocial events and biological processes relevant to colon cancer [19].

Common physiological responses to stress may influence the colon cancer process. The responses to stressors involve subjective perceptions of threat and subsequent activation of the autonomic nervous system and the hypothalamic–pituitary–adrenal axis. Catecholamines, glucocorticoids and other stress hormones are subsequently released from the adrenal gland, brain and sympathetic nerve terminals and can modulate the activity of multiple components of the tumor microenvironment. Effects can include the promotion of tumor-cell growth, migration and invasive capacity, and stimulation of angiogenesis by inducing production of pro-angiogenic cytokines. Stress hormones can also activate oncogenic viruses and alter several aspects of immune function, including antibody and cytokine production and cell trafficking. Collectively, these downstream effects create a permissive environment for tumor initiation, growth and progression [20-22].

The impact stressful life events on biological precursors of colon cancer such as colon polyps, however, has yet to be determined. Also, no study to date has explored the influence of stressful life events on colon polyps among African Americans, a group known to experience a high burden of colon cancer as well as increased reports of life stress. For example, it has been reported that African Americans report more chronic stress stemming from various sources including perceived discrimination and neighborhood stress [23]. Understanding how stress impacts colon polyps will inform researchers and clinicians on the importance of providing psychosocial interventions during the pre-cancerous stage.

The current study seeks to investigate the association between stressful life events and the presence of colon polyps among African-American men and women. Specifically, this study will explore whether stressful life events are associated with the presence of colon polyps, the number of colon polyps and severity of colon polyps, as well as compare reports of stressful life events among African-American participants seeking colon cancer screening, follow-up and diagnostic evaluation.

## Methods

### Patient recruitment

African American patients (self-identified) coming to a university teaching hospital in the mid-Atlantic region for colonoscopy were recruited from June 2011 to October 2011 in a cross-sectional sampling. The study was approved by the Institutional Review Board of Howard University. Written informed consent was obtained after explaining the study. An interview conducted by a research assistant gathered data about their socio demographics including education, family medical history, alcohol and smoking exposure, personal medical history and personal health habits. Patients with HIV, Hepatitis B, Hepatitis C, bleeding disorders and history of colon cancer were not included in the study. Colonoscopies where the endoscopist was able to reach the cecum were considered complete. Incomplete colonoscopies were excluded from analysis. Patients in which no polyps were found during colonoscopy were considered normal.

Specimens of the patients with biopsy or polypectomy were sent to pathology and were evaluated by a gastrointestinal pathologist. The location and number of the polyps were recorded during colonoscopy. The polyps were classified as hyperplastic polyps, tubular adenomas, tubulovillous adenoma, or villous adenomas based on histology.

Patients who had a colonoscopy for the first time and were asymptomatic were considered screening colonoscopy participants. Patients who had earlier normal colonoscopy or polyps removed and came for follow-up were called follow-up colonoscopies. Patients undergoing colonoscopies for symptoms like abdominal pain or bleeding per rectum were grouped under diagnostic colonoscopies.

### Assessment of life events

Life events were assessed prior to colonoscopy by the Life Events Questionnaire [24]. The LEQ is an 82-item self-report survey that lists common life events (e.g., Health, work, residence, Marriage, family). Respondents indicated whether they experienced each event during the past year. For events that were endorsed, participants classified the event as “good” or “bad” and rated the severity on a scale of 0–3 where 0 = no effect and 3 = great effect. A positive events score was the sum of the all ratings marked as good by the patient. A negative events score was the sum of the all ratings marked as bad by the patient. A total events score was the sum of positive and negative events.

### Statistical analysis

Patients who completed the socio demographic questionnaire, life events questionnaire and had a complete colonoscopy were included in analysis. Descriptive statistics were conducted using median scores and percentages. Additional analyses were conducted using KrusKal-Wallis nonparametric test. We developed a linear regression model to assess the predictor of Life Events score. In each model we introduced age, gender, BMI, education, personal history of other disease into model and built the final model with a backward stepwise approach. Statistical analyses were done using STATA (StatCorp, College Station, TX). P < 0.05 was considered significant.

#### Sample size consideration

We postulated that the difference between patients with colorectal polyp and controls in total Life Events score is 10 (Standard Deviation = 20). A sample size of 60 controls and 60 patients with polyp provide a power of 0.8 to detect this difference significantly (a = 0.05, two-sided).

## Results

### Participant characteristics

One hundred and ten patients were included in the final analysis (Table 1). Ages ranged from 33 to 82 with a mean of 58.3 (SD = 8.87). The study population included 59% (n = 65) females and 41% (n = 45) males. One hundred six participants (97%) had at least a high school education. Forty two (39%) patients were overweight (BMI ≥25) and 39% (n = 42) were obese (BMI ≥30). Eight two (75%) participants had history of other medical conditions including hypertension, diabetes, depression or asthma. Additional participant characteristics are shown in Table 1.

**Table 1:** Patient characteristics by pathology diagnosis

	**Normal**	**Hyperplastic polyp**	**Adenoma**	**P value**
	**n = 43**	**n = 23**	**n = 44**	
**Age, mean (SD)**	59 (10.5)	58 (9.2)	58 (6.8)	0.9
**Gender**				0.9
Male	19 (44%)	9 (40%)	17 (39%)	
Female	24 (56%)	14 (60%)	27 (61%)	
**Education**				0.02
Middle school	1 (2%)	2 (9%)	0	
High school	25 (60%)	5 (22%)	21 (48%)	
College & higher	16 (38%)	16 (70%)	23 (52%)	
**Indication**				0.6
Screening	21 (49%)	14 (61%)	28 (64%)	
Follow-up	15 (35%)	5 (22%)	10 (23%)	
Diagnostic	7 (16%)	4 (17%)	6 (14%)	
**BMI**				0.8
<18.5	0	0	1 (2%)	
18.5-24.9	12 (28%)	5 (23%)	7 (16%)	
25.0-29.9	14 (33%)	10 (45%)	18 (41%)	
30.0-34.9	11 (26%)	5 (23%)	10 (23%)	
= > 35.0	6 (14%)	2 (9%)	8 (18%)	
Medical history of any other disease	32 (74%)	14 (61%)	36 (82%)	0.2
Family history of cancer	17 (40%)	10 (43%)	18 (41%)	0.9
Family history of colon cancer	4 (9%)	3 (13%)	11 (25%)	0.1
**Smoking status**				0.3
Current smokers	7 (16%)	3 (13%)	14 (32%)	
Previous smokers	15 (35%)	8 (35%)	14 (32%)	
**Alcohol consumption**				0.04
Yes	25 (58%)	7 (30%)	15 (36%)	
No	18 (42%)	16 (70%)	27 (64%)	

### Life events scores in patients with polyps vs. patients without polyps

**Figure 1:**
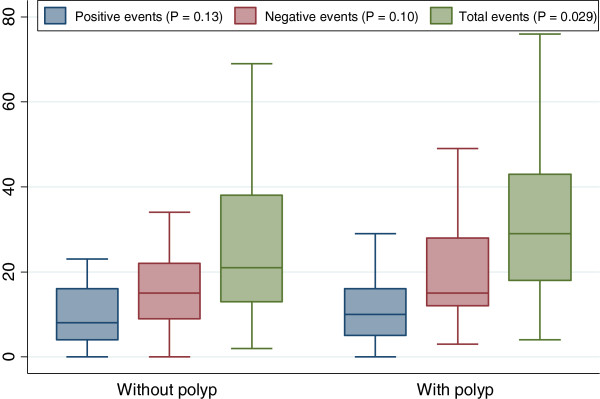
Distribution of positive, negative and total life events score by polyp status.

Total life events scores were higher in patients with polyps (Median = 29) compared to patients without polyps (Median = 21; P = 0.029); Figure 1. There were no significant differences between participants with and without colon polyps on negative life events scores or positive life events scores.

### Life events scores association in normal vs. adenoma

**Figure 2:**
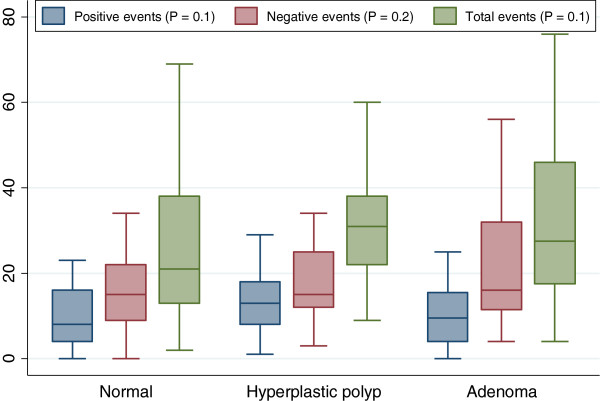
Distribution of positive, negative and total life events score by pathologic results.

Total, positive or negative life events did not differ significantly between normal and adenoma participants (Figure 2).

### Life Events scores in screening vs. follow-up vs. diagnostic patients

**Figure 3:**
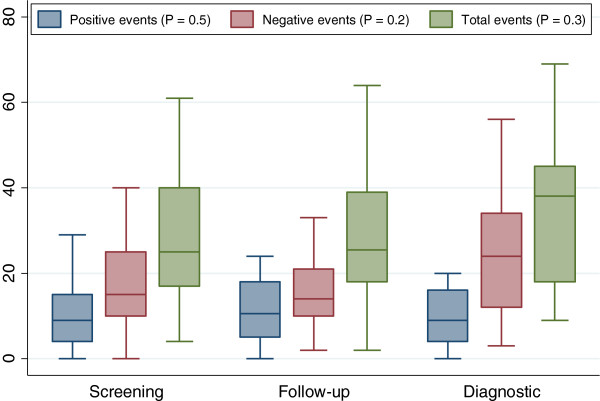
Distribution of life positive, negative and total life events score by colonoscopy indication.

An analysis of screening, follow-up and diagnostic patients in relation to total, positive and negative life events showed that total, positive or negative life events did not differ significantly between different indications (Figure 3).

### Predictors of life events score

A linear regression analysis indicated that male gender was associated with 9.0 unit lower total Life Events score (95% CI = −17 - −2, P = 0.025). Age, education, BMI, medical history of disease were not significantly associated with total Life Events scores.

Increasing age (beta = −0.4, P = 0.017) and male gender (beta = −5.7, P = 0.031) were associated with lower negative Life Events scores. Patients with medical history of any disease (beta = 6.0, P = 0.047) had higher negative Life Events score. We did not find any significant predictor for positive life events score.

## Discussion

As previously noted, African Americans have an increased prevalence of colon cancer compared with Non-Hispanic Whites in the US [4]. Several lifestyle factors have been implicated as risk factors for colon cancer. Psychosocial stress has recently been included as a potential risk factor for colon cancer development. Before this investigation, no study had examined relationships of life events on colon polyps, a precursor of colon cancer. Also, no other study had examined this question in African American patients.

We found that total life events scores were higher in patients with polyps compared to patients without polyps. This finding suggests that not only negative life events but also events rated as positive influence the presence of colon polyps. Most Life Events research has focused on the effect of negative events on health outcomes, however this finding shows the importance of acknowledging general stress appraisal and its impact on health.

Colon polyp development involves genetic and epigenetic changes and environmental effectors such as stress in this process can drive the normal colonic epithelial cells to hyperplastic and adenomas [25-27]. Stress can induce hormonal release such as catecholamines and glucocorticoids from adrenal gland and cause alteration of cell proliferation, In addition, stress hormones can make the human cell prone to infections due to the alteration of immune system, may activate oncogenic viruses and alter several aspects of immune function, including antibody production, cytokine production profiles and cell trafficking. Hence, stress can affect cell growth rate and hence drive normal cells to malignancy due to permissive microenvironment conditions. Corticotrophin-releasing factor; interleukin-6; matrix metalloproteinase; vascular endothelial growth factor play important roles in colon carcinogenesis [21,28]. These stress related factors may influence colon polyp development [20,22].

Persons reporting increased levels of stress have also reported increased smoking, poor diet and low levels of physical activity [29,30]. Each of these factors have been associated with colon polyp development. For example, Burnett-Hartman et al. (2011) found that consumption of charred meats and heavy cigarette smoking was positively associated with colorectal polyps [29]. In a related study, Karagianni et al. (2010) found that increased physical activity was associated with decreased colon polyp presence [30]. This information, taken together, suggests that experiencing total (including stress) life events could induce the adoption of certain unhealthy behaviors that may in turn promote colon polyp development.

A major strength of this study is that it has examined a novel research question, the association of total life events and colon polyps in African-American participants. Our findings suggest the possibility that total life events are a risk factor for colon polyps thus adding to the evidence that psychosocial factors play a role in the pre-clinical cancer process. Another strength of this study is the use of the Life Events Questionnaire. Many life events questionnaires require participants to select from a list of pre-determined events without giving them the option to rate the relative impact of the event. The Life Events Questionnaire used in this study presents an extensive list of events while allowing the participant to rate the overall magnitude as well whether the event had a positive or negative impact.

One limitation of this study is the cross-sectional nature of the study design. As a result, our ability to infer causal relationships between life events and colon polyps is restricted. Future studies could address this issue by assessing a group of polyp free individuals at an early stage of life while tracking their stressful life events exposure and colon polyp development over time. Another limitation of this study is that while the study addresses an understudied population, African-Americans, there are other ethnic groups that could be explored as well. It would be beneficial to understand if the same or different relationships exist between total (positive and negative) life events and colon polyp development in other ethnic populations.

## Conclusion

We found that total life events were associated with the presence of colon polyps in this group of African Americans who were participating in a colon screening program and we did not find any difference between different polyp pathology. This study adds to the growing body of evidence linking psychosocial stress to the colon cancer process. Life events could have happened after polyp formation and this study cannot confirm the causal relationship. Future studies can assess the causality between life stress and polyp formation by employing a prospective research design and additional ethnic populations.

## Competing interests

The authors declare that they have no competing interests.

## Authors’ contributions

HA, TM, CW, CLH, and AOL participated in the design of the study and data analysis. HA and SMN drafted the manuscript. TT, HB, HH, BS, and CW were involved in the completing the questioners. HH and AOL recruited the patients. MN performed the statistical analysis. All authors read and approved the final manuscript.

## Pre-publication history

The pre-publication history for this paper can be accessed here:

http://www.biomedcentral.com/1471-230X/13/101/prepub
